# Population pharmacokinetics and optimized dosing of piperacillin-tazobactam in hematological patients with febrile neutropenia

**DOI:** 10.1128/aac.01253-25

**Published:** 2025-11-25

**Authors:** Julia Laporte-Amargos, Marta Ulldemolins, María Patricia Hernández-Mitre, Jason A. Roberts, Raul Rigo-Bonnin, Francisco Carmona-Torre, Maria Huguet, Pedro Puerta-Alcalde, Montserrat Arnan, Jose Luis del Pozo, Anna Torrent, Carolina García-Vidal, Anna Sureda, Alba Bergas, Enric Sastre-Escolà, Jordi Carratalà, Carlota Gudiol

**Affiliations:** 1 Department of Infectious Diseases, Bellvitge University Hospital 16383, L'Hospitalet de Llobregat, Barcelona, Spain; 2 School of Medicine, University of Barcelona 16724, Barcelona, Spain; 3 UQ Centre for Clinical Research, Faculty of Health, Medicine, and Behavioural Sciences (HMBS), The University of Queensland 1974, Brisbane, Queensland, Australia; 4 Herston Infectious Diseases Institute (HeIDI), Metro North Health 157827 , Brisbane, Australia; 5 Department of Pharmacy, Royal Brisbane and Women's Hospital 3883, Brisbane, Australia; 6 Department of Intensive Care Medicine, Royal Brisbane and Women’s Hospital, Brisbane, Australia; 7 Division of Anesthesia Critical Care and Emergency and Pain Medicine, Nimes University Hospital, UR UM 103, University of Montpellier, Nimes, France; 8 Department of Clinical Laboratory, Bellvitge University Hospital, Bellvitge Biomedical Research Institute (IDIBELL) 16383, L'Hospitalet de Llobregat, Barcelona, Spain; 9 Department of Infectious Diseases, Clínica Universidad de Navarra, Navarra Institute for Health Research (IdiSNA) 16755, Pamplona, Navarra, Spain; 10 Department of Clinical Haematology, Institut Català d’Oncologia-Badalona, Hospital Germans Trias i Pujol, Josep Carreras Leukaemia Research Institute 16514 , Badalona, Spain; 11 Infectious Diseases Department, Institut d'Investigacions Biomèdiques August Pi i Sunyer (IDIBAPS), Hospital Clínic de Barcelona 16493, Barcelona, Spain; 12 Department of Clinical Haematology, Institut Català d’Oncologia-Hospitalet, IDIBELL 115336, L’Hospitalet de Llobregat, Barcelona, Spain; 13 Centro de Investigación Biomédica en Red de Enfermedades Infecciosas (CIBERINFEC), Instituto de Salud Carlos III 38176, Madrid, Spain; 14 Bellvitge Biomedical Research Institute (IDIBELL) 115336, L’Hospitalet de Llobregat, Barcelona, Spain; 15 Institut Català d’Oncologia 16529, L'Hospitalet de Llobregat, Barcelona, Spain; Providence Portland Medical Center, Portland, Oregon, USA

**Keywords:** febrile neutropenia, piperacillin-tazobactam, extended infusion, continuous infusion, pharmacokinetics, pharmacokinetics/pharmacodynamics, creatinine clearance

## Abstract

Hematological patients with febrile neutropenia receiving piperacillin-tazobactam may experience pharmacokinetic alterations that compromise drug exposure. We aimed to characterize the population pharmacokinetics of piperacillin in plasma and provide optimized dosing recommendations for this patient population. A population pharmacokinetic analysis was conducted in patients who received piperacillin-tazobactam as part of the BEATLE study, which compared the efficacy, safety, and pharmacokinetic/pharmacodynamic target attainment of β-lactams administered in extended infusion versus short 30 min infusion in adult hematological patients with febrile neutropenia. Monte Carlo simulations were performed to evaluate, for each dosing regimen, the probability of attaining (i) an efficacy target of unbound piperacillin concentrations above the minimum inhibitory concentration (MIC) of the bacteria for the entire dosing interval (100% ƒ*T*_>MIC_), and (ii) a toxicity threshold of ≥160 mg/L. A total of 44 patients and 221 plasma concentrations were included. A one-compartment model best described piperacillin plasma pharmacokinetics, with Cockcroft-Gault creatinine clearance (CrCL) significantly influencing drug clearance. Dosing simulations showed that extended and continuous infusions were superior to short 30 min infusions in the attainment of 100% ƒ*T*_>MIC_, even for bacteria with low to intermediate MIC (≤2–4 mg/L). In patients with higher CrCL (>90 mL/min) or infections caused by less susceptible Gram-negative bacilli (MIC: 8–16 mg/L), only continuous infusions of 12–16 g/day were likely to achieve 100% ƒ*T*_>MIC_. These findings support the use of extended or continuous infusions of piperacillin in the initial management of patients with febrile neutropenia, particularly in patients with higher CrCL or when infections caused by less susceptible pathogens, such as *Pseudomonas aeruginosa*, are suspected.

## INTRODUCTION

Piperacillin-tazobactam is frequently used as empirical treatment for febrile neutropenia, in accordance with current international clinical guidelines (1, 2). As a β-lactam antibiotic, piperacillin-tazobactam exhibits optimal bactericidal activity when free (unbound) drug concentrations are maintained above the minimum inhibitory concentration (MIC) of the bacteria for a specific percentage of the dosing interval (% ƒ*T*_>MIC_) (3). In the treatment of patients with severe infections, the β-lactam pharmacokinetic-pharmacodynamic (PK/PD) target associated with the best clinical outcomes remains debated, but emerging evidence suggests that a 100% ƒ*T*_>MIC_ may be necessary for the treatment of severe infections (4–6). This 100% ƒ*T*_>MIC_ may also be necessary for the treatment of hematological patients with febrile neutropenia even in the absence of sepsis, as their profoundly impaired immune response limits its contribution to antibiotics to control the infection (7). Moreover, a more demanding target of unbound concentrations above 4× MIC for the entire dosing interval (100% ƒ*T*_>4×MIC_) may prevent the emergence of resistance and facilitate a higher exposure in high-inoculum, deep-seated infections (8, 9).

However, major pathophysiological changes such as fluid shifts and a hyperdynamic state are observed in patients with febrile neutropenia in the context of malignancy, medical management, and fever. These alterations may lead to profound variations in the PK of hydrophilic drugs like β-lactams, notably affecting both volume of distribution (Vd) and clearance (CL) even in the absence of sepsis or septic shock (7). As a result, piperacillin concentration-time profiles may be altered and the attainment of optimal antibiotic exposures may be compromised, particularly against less susceptible Gram-negative bacilli with higher MIC (7, 10).

The aim of this study was to describe the population PK of piperacillin in patients with hematological malignancies and febrile neutropenia treated with piperacillin-tazobactam in order to provide optimized dosing recommendations for this complex patient population.

## MATERIALS AND METHODS

### Study design, setting, and participants

We included a subset of patients included in the BEATLE study who received piperacillin-tazobactam as empirical antibiotic treatment for febrile neutropenia. The BEATLE study was a multicenter randomized controlled trial (RCT) that compared the efficacy, safety, and PK/PD target attainment of piperacillin-tazobactam, cefepime, and meropenem administered as extended infusion (3–4 h infusion) versus standard 30 min infusion in hematological patients with febrile neutropenia (11).

The BEATLE study was conducted between November 2019 and June 2022 in four Spanish university hospitals. Inclusion criteria were as follows: (i) adult patients undergoing chemotherapy for acute leukemia or hematopoietic stem cell transplantation; (ii) presence of febrile neutropenia (defined as axillary temperature ≥ 38.0°C and <500 neutrophils/mm^3^ or <1,000 expected to drop within 24–48 h); and (iii) indication for empirical therapy with one of the study antibiotics. Exclusion criteria included known allergy to the study antibiotics, systemic antibiotic therapy at febrile neutropenia onset, epilepsy, severe renal impairment (defined as estimated glomerular filtration rate [eGFR] < 30 mL/min/1.73 m^2^ by the Chronic Kidney Disease Epidemiology Collaboration [CKD-EPI] equation), and prior enrollment in the study without resolution of the first episode or enrollment 5 weeks before the new febrile neutropenic episode. All patients or their legal representatives provided written informed consent to participate in the study. The protocol and primary outcomes of the BEATLE RCT have been published elsewhere (11, 12).

### Antibiotic dosing, data collection, and blood sampling

The study protocol specified piperacillin-tazobactam dosing as follows: patients with an eGFR > 40 mL/min/1.73 m^2^ (estimated by CKD-EPI equation) received 4 g/0.5 g of piperacillin-tazobactam every 6 h (q6h), while those with eGFR between 30 and 40 mL/min/1.73 m^2^ received 4 g/0.5 g every 8 h (q8h). At the onset of febrile neutropenia, all patients received the first dose as a short 30 min infusion. In the extended infusion group, subsequent doses were administered over a period equal to half the dosing interval. In the intermittent infusion group, piperacillin-tazobactam continued to be administered over 30 min.

Demographic and clinical data were collected at inclusion and on the days of sampling. Plasma samples were collected during the first 5 days of treatment on three occasions via a peripheral catheter used exclusively for sampling. On two occasions, samples were collected at −10 min (*C*_min_) and 180 min after the start of infusion. On the third sampling occasion, a sample was taken at 270 min in addition to the *C*_min_ and 180 min samples. As a pre-specified intensive PK sub-study, a subgroup of patients underwent intensive PK sampling over one dosing occasion. For patients treated with extended infusion, samples were collected at *C*_min_, 180 (end of the infusion), 210, 240, and 270 min; for patients treated with a short 30 min infusion, samples were collected at *C*_min_, 30, 60, 90, 180, and 270 min. Plasma samples were immediately processed after collection and stored at –80°C until bioanalysis.

Piperacillin total plasma concentrations were measured using a validated ultra-high-performance liquid chromatography method coupled with tandem mass-spectrometry (13). Lower limit of quantification, measuring interval, inter-day imprecision, absolute relative biases, normalized-matrix factors, and normalized recoveries were 0.58 mg/L, (0.58–175 mg/L), ≤8.9%, ≤5.0%, (100.2%–103.3%), and (99.4%–106.5%), respectively. No carryover or interferences were observed.

### Population pharmacokinetic analysis

Population PK modeling was performed using Monolix 2024R1 (Lixoft SAS, a Simulations Plus company), which implements the stochastic approximation expectation maximization algorithm (14).

### Basic model building, covariate analysis, and model diagnostics

Initially, one- and two-compartment models with first-order elimination were evaluated. All individual parameters were assumed to be log-normally distributed, and between-subject variability (BSV) and between-occasion variability (BOV) were described using an exponential model. Constant, proportional, and combined error models were tested.

From the base model, the influence of the following covariates on piperacillin PK parameters was explored: age, sex, height, weight at admission, eGFR (15) and creatinine clearance (CrCL) estimated by the Cockcroft-Gault equation (16) at each sampling time, bilirubin plasma concentrations, albumin and total protein serum concentrations, and baseline severity scores (Acute Physiology and Chronic Health Evaluation [APACHE] II (17), Sepsis-Related Organ Failure Assessment [SOFA] (18), and Multinational Association for Supportive Care in Cancer [MASCC] [19]). Continuous and categorical covariates were individually added to the base model in the forward inclusion step until there was no drop in the −2log-likelihood (−2LL) over 3.84 (*P* < 0.05). In the backward elimination step, the covariates were removed from the model one by one, and the effect of each removal on the global fit was statistically tested. The covariates were kept in the final model if the backward elimination caused an increase in −2LL of at least 10.83 (*P* < 0.001), and the effect of the covariate on the PK parameter was biologically plausible. Model selection and evaluation were based on the Akaike information criterion, the corrected Bayesian information criterion, the relative standard error (RSE) of the fixed and random effects, and goodness-of-fit (GOF) plots (20). A prediction-corrected visual predictive check (pc-VPC) was performed using 500 simulations with the final model (21). Non-parametric bootstrapping (*n* = 1,000) was conducted to assess the stability and robustness of parameter estimates and to derive 95% confidence intervals. A detailed description of model development is provided in the Supplemental Material.

### Monte Carlo dosing simulations and probability of target attainment

Monte Carlo dosing simulations were performed with the final covariate model using Simulx 2024R1 (Lixoft SAS, a Simulations Plus company). A total of 1,000 individual PK profiles over the first 48 h of treatment were simulated for the following regimens: (i) 4 g in a 30 min infusion q6h or q8h; (ii) 4 g in a 3 h extended infusion q6h with and without a 2 g loading dose (30 min infusion), and 4 g in a 4 h extended infusion q8h with and without loading dose; (iii) 2 g loading dose followed by continuous infusions of 8, 12, and 16 g over 24 h. When a loading dose was simulated, the maintenance regimen commenced immediately after the end of the short infusion. Unbound piperacillin concentrations were calculated assuming a 30% protein binding (22).

Optimal piperacillin exposure was defined considering efficacy and toxicity targets. The primary efficacy target was set as maintaining unbound concentrations above the MIC for the entire dosing interval (100% ƒ*T*_>MIC_) for the most likely Gram-negative bacilli, including *Pseudomonas aeruginosa*. A more stringent secondary target of 100% ƒ*T*_>4×MIC_ was also considered, as this higher exposure may be required for efficacy as well as for preventing the emergence of intra-treatment resistance in deep-seated, high-inoculum Gram-negative infections such as nosocomial pneumonia (8, 9). The toxicity threshold was set as unbound concentrations ≥ 160 mg/L for the entire dosing interval, based on published retrospective evidence that associated piperacillin trough (*C*_min_) or average steady-state concentrations in continuous infusion (*C*_ss_) values between 157 and 450 mg/L with a higher incidence of toxicity (23–25). Additionally, from an efficacy perspective, unbound concentrations exceeding 10-fold the European Committee on Antimicrobial Susceptibility Testing (EUCAST) susceptibility breakpoint for *P. aeruginosa* (160 mg/L) were considered unlikely to provide additional benefit. Therefore, for each dosing regimen, the probability of target attainment (PTA) was calculated for various unbound trough (ƒ*C*_min_) or unbound average steady-state concentrations in continuous infusion (ƒ*C*_ss_), considering the EUCAST clinical breakpoint and epidemiological cut-off values (ECOFF) for Enterobacterales and *P. aeruginosa* in combination with tazobactam (8 and 16 mg/L, respectively). The optimal PTA was defined as ≥90% for all analyses.

## RESULTS

**Fig 1:**
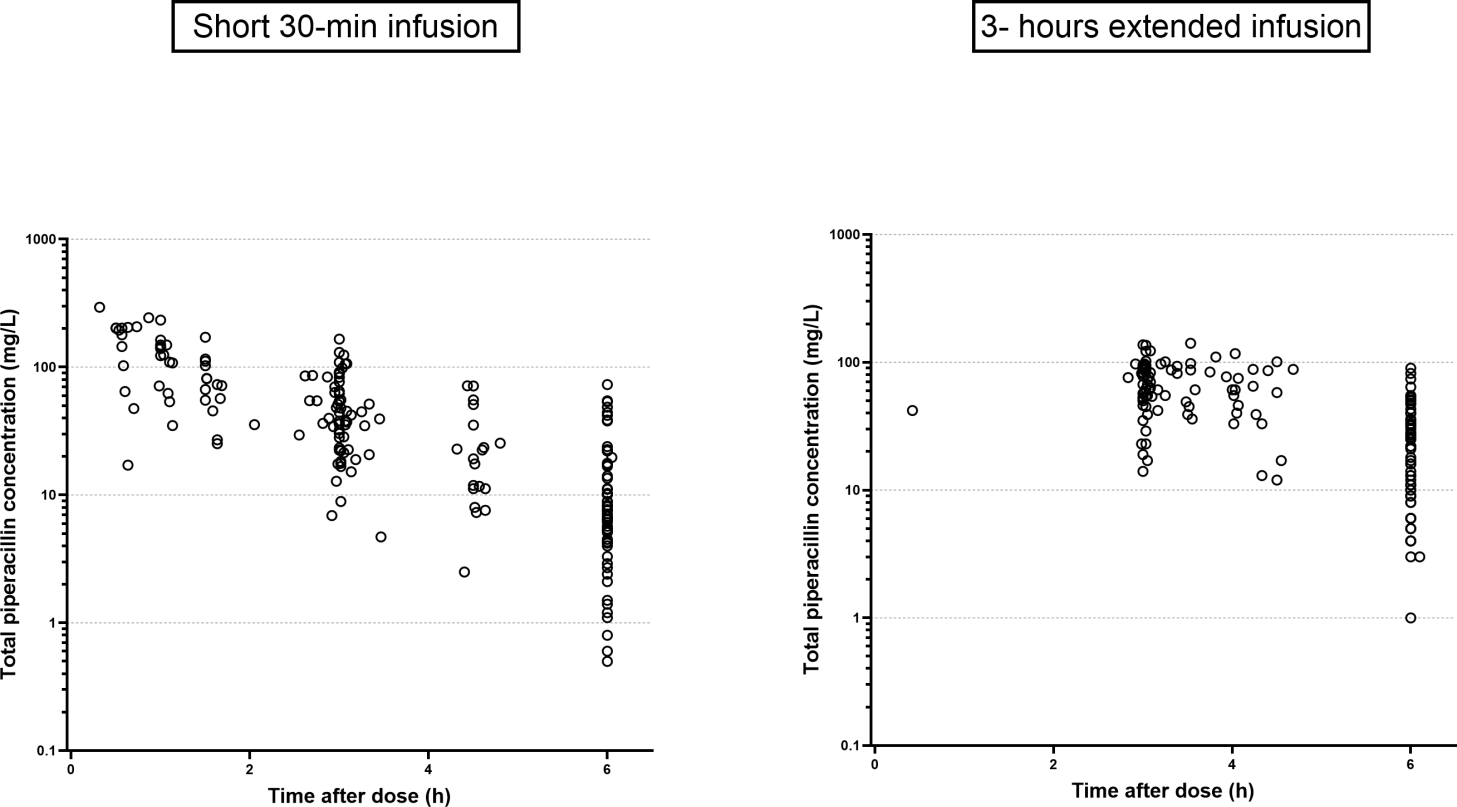
Observed concentrations over time (after dose) of piperacillin in patients treated with short 30 min infusion (left) or 3 h extended infusion (right). Concentrations are in mg/L, time is in h, and the *Y*-axis is in logarithmic scale.

Forty-four patients were included in the study; Table 1 summarizes their demographic and clinical characteristics. Twenty-two patients (50%) had a CrCL > 90 mL/min, and only three experienced hypotension at febrile neutropenia onset without requiring vasoactive drugs. Thirteen patients (29.5%) had a microbiologically documented infection, mainly due to Enterobacterales (*n* = 6) and *P. aeruginosa* (*n* = 3) (Table S1). Piperacillin plasma concentration-time profiles by group are presented in Fig. 1.

**TABLE 1:** Demographic and clinical characteristics of the patients included in the study*^a^*^,^*^b^*

	*N* = 44 (%)
Sex (male)	21 (47.7)
Age (years; mean, SD)	55.4 (10.2)
Weight (kg; median, IQI)	70 (62.4–77.6)
Height (cm; mean, SD)	165.6 (10.8)
BMI (kg/m^2^; median, IQI)	26.3 (24.1–28.0)
Underlying hematological malignancy	
Acute myeloid leukemia/myelodysplastic syndrome	9 (20.5)
Lymphoma	13 (29.5)
Multiple myeloma	14 (31.8)
Acute lymphoblastic leukemia	3 (6.8)
Other	5 (11.4)
Reason for hospital admission	
Treatment for acute leukemia	4 (9.1)
Hematopoietic stem cell transplant	40 (90.9)
Autologous	27/40 (67.5)
Allogenic	13/40 (32.5)
High risk MASCC score (<21)^*c*^	20 (45.5)
SOFA score; mean (SD)^*c*^	5 (1.6)
APACHE II score; mean (SD)^*c*^	18.0 (3.5)
Neutrophils ≤ 100/mm^3^; median (IQI)^*c*^	0 (0–5)
Estimated glomerular filtration rate (mL/min/1.73 m^2^) CKD-EPI equation; mean (SD)^*c*^	105.8 (28.6)
Creatinine clearance (mL/min) Cockcroft-Gault; median (IQI)^*c*^	96.2 (83.4–125.7)
Creatinine clearance in the extended infusion group	93.6 (83.1–134.9)
Creatinine clearance in the intermittent infusion group	99.8 (84.7–117)
Albumin (g/dL); mean (SD)^*c*^	3.4 (0.4)
Protein (g/dL); mean (SD)^*c*^	5.6 (0.5)
Bilirubin (mg/dL); median (IQI)^*c*^	0.62 (0.47–0.94)
Diuresis last 24 h prior to the day of PK sampling (mL/day); mean (SD)^*c*^	2,177 (999)
Hypotension^*c*^^,^^*d*^	3 (6.8)
Source of infection	
Mucositis grades III–IV	15 (34.1)
Neutropenic enterocolitis	1 (2.3)
Other abdominal source	7 (15.9)
Intravascular catheter	1 (2.3)
Fever of unknown origin	20 (45.5)
Piperacillin-tazobactam dose and infusion time	
4 g q6h in 3 h extended infusion	21 (47.7)
Subgroup of patients that underwent intensive PK sampling	10/21 (47.6)
4 g q6h in 30 min intermittent infusion	23 (52.3)
Subgroup of patients that underwent intensive PK sampling	14/23 (60.9)
*C*_min_ of piperacillin (mg/L) in 3 h extended infusion; median (IQI)	19.9 (8.8–33.2)
*C*_50_ of piperacillin (mg/L) in 3 h extended infusion (end of the infusion); median (IQI)	72.6 (53.8–91.0)
*C*_min_ of piperacillin (mg/L) in 30 min intermittent infusion; median (IQI)	7.6 (4.5–17.7)
*C*_50_ of piperacillin (mg/L) in 30 min intermittent infusion; median (IQI)	38.0 (23.3–63.3)
Intensive care unit admission	1 (2.3)
Clinical resolution on day 5	37 (84.1)
30-day all-cause mortality	1 (2.3)

^*a*^ Continuous variables are expressed as mean (SD, standard deviation) or median (IQI, interquartile interval), as appropriate based on their distribution.

^*b*^ BMI, body mass index; APACHE II: Acute Physiology and Chronic Health Evaluation II; *C*_min_, pre-dose concentration of piperacillin; *C*_50_, concentration of piperacillin at 180 min after starting the administration of the antibiotic.

^*c*^ At the onset of febrile neutropenia.

^*d*^ Defined as systolic/diastolic blood pressure < 90/60 mmHg; or a decrease in the systolic blood pressure of 30 mmHg or greater; or a decrease in the mean arterial pressure of 20 mmHg or greater.

### Population PK analysis

**Fig 2:**
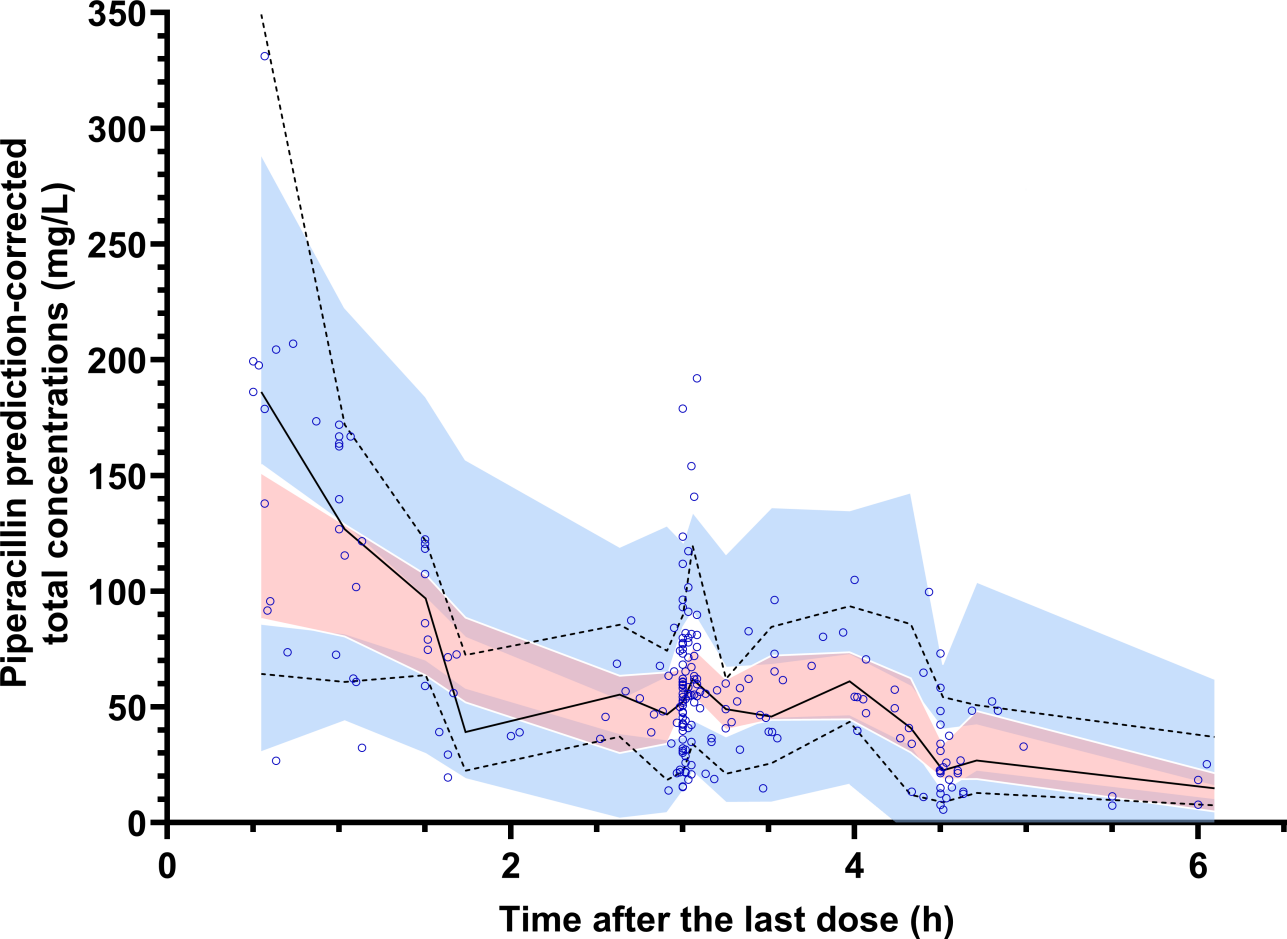
pc-VPC for the final model for piperacillin. Blue dots represent the observed concentrations over time data. Dotted black lines represent the 5th and 95th empirical percentiles of observed data, and solid black lines represent the 50th empirical percentile of observed data. Shaded light blue areas are the 95% prediction intervals of the 5th and 95th percentiles, and the shaded pink area is the 50th prediction interval.

Two hundred and twenty-one blood samples from 122 dosing occasions were collected for PK analysis. A one-compartment model with first-order elimination was the structural model that best fitted the concentration-time data. Although visual inspection initially suggested that a two-compartment model might also be appropriate, estimation of the central and peripheral Vd was biased, likely due to several patients receiving extended infusions and the majority of samples being collected during the elimination phase (i.e., from the midpoint of the dosing interval onward). Regarding the covariate analysis, time-varying CrCL was the only covariate that significantly influenced CL, reducing the BSV on CL by 18.6%. Importantly, its effect on BOV was negligible, likely due to minimal intra-subject variation in CrCL between occasions (median variation of 6.6% between the first and subsequent sampling occasions; interquartile range: 3.6%–14.4%). The relationship between CL and CrCL was described by the following equation:


$${CL}_{i}\left(L/h\right)=12\times {\frac{{CrCL}_{i}}{99.3}}^{0.64},$$

where CL is the individual CL in L/h and CrCL = CrCL in mL/min estimated with the Cockcroft-Gault equation at each sampling time, normalized to the median CrCL of our patient population (99.3 mL/min). The final PK model and the parameter estimates are summarized in Table 2. The pc-VPC and the GOF plots for the final model are shown in Fig. 2 and Fig. S1, respectively.

**TABLE 2:** Population pharmacokinetic parameter estimates for piperacillin and bootstrap results (*n* = 1,000)^*a*^

Parameter	Estimate (% RSE) [Shrinkage %]	Median bootstrap estimates (percentiles 2.5%–97.5%)
Fixed effects		
CL (L/h)	12.0 (6.2%) [16.5%]	12.0 (10.8–13.6)
Vd (L)	29.8 (10.9%) [18%]	29.7 (23.4–37.9)
CrCL effect on CL	0.64 (27.2%)	0.62 (0.23–0.91)
Random effects		
BSV CL (CV %)	36.2 (14.9%)	36.1 (21.2–50.9)
BSV Vd (CV %)	57.3 (15.2%)	56.9 (35.0–79.5)
BOV CL (CV %)	16.4 (23.5%)	14.1 (8.2–23.3)
Residual variability		
a (constant) (mg/L)	5.5 (19.3%)	5.5 (0.15–10.5)
b (proportional)	0.20 (11.1%)	0.21 (0.10–0.26)

^*a*^ BSV, between-subject variability expressed as coefficient of variation (CV %); BOV, between-occasion variability expressed as coefficient of variation (CV %); a, constant component of the residual error; b, proportional component of the residual error.

### Monte Carlo dosing simulations and probability of target attainment

Monte Carlo dosing simulations were performed for different dose regimens using the final PK model for piperacillin, fixing different values of CrCL within the 60–150 mL/min range, consistent with the range observed in our population (Table 3). This approach was chosen to facilitate comparison across representative values of CrCL. Dosing simulations showed that, during the first 48 h of therapy, extended and continuous infusion regimens following a 2 g loading dose were superior to short 30 min infusions for the early attainment of the 100% ƒ*T*_>MIC_ target, even for lower to intermediate MICs (≤2–4 mg/L). In patients with higher CrCL (>90 mL/min) or infections caused by less susceptible Gram-negative bacilli (MIC: 8–16 mg/L), continuous infusions of 12–16 g/day following a 2 g loading dose were the only regimens likely to achieve 100% ƒ*T*_>MIC_. Table 4 provides optimized dosing recommendations for empirical treatment for this population stratified by CrCL and target ƒ*C*_min_ or ƒ*C*_ss_. Of note, we do not recommend daily doses below the minimum values stated in the product information for any CrCL range, even if the simulations suggest sufficient PTA in plasma.

**TABLE 3:** PTA (%) of maintaining an optimized piperacillin exposure (100% ƒ*T*_>MIC_) per dosing regimen for the first 48 h of treatment*^a^*^,^*^b^*

Target ƒ*C*_min_ or ƒ*C*_ss_ (mg/L)																	
CrCL	0.5	1	2	4	8	16	32	64	CrCL	0.5	1	2	4	8	16	32	64
*4 g q8h 30 min infusion*									*4 g q6h 30 min infusion*								
60 mL/min	93.3	89.5	82.6	72.6	53.4	24.2	1.3	0.6	60 mL/min	97.3	95.8	93.7	88.2	76	49.5	8.1	0
90 mL/min	84.3	77.4	68.2	54.4	33.2	8.5	0.3	0.3	90 mL/min	94.4	91.5	85.3	75.2	58.3	26.7	1.9	0
120 mL/min	70.9	64.4	51.7	38.7	22	4.6	0	0	120 mL/min	86.1	80.2	71.6	58.8	40.5	15.2	0.3	0
150 mL/min	67.5	58.6	47	31.6	17.5	1.8	0	0	150 mL/min	82.7	76.5	67.9	53.7	34	10.9	0.2	0
*4 g q8h 4 h infusion*									*4 g q6h 3 h infusion*								
60 mL/min	97.8	96.8	94.2	86.9	49.8	4.3	0	0	60 mL/min	99.4	99.1	97.7	95.8	79.9	23.9	0.6	0
90 mL/min	95.7	92.8	86.9	74.2	34.8	2.2	0	0	90 mL/min	98.7	97.5	95.8	90.5	66.5	14.8	0.2	0
120 mL/min	88.5	82.1	73.4	58	20.5	0.4	0	0	120 mL/min	96.7	93.1	88.5	77.8	52.8	6.3	0	0
150 mL/min	85	79.7	69.6	52.8	15.6	0.2	0	0	150 mL/min	93.4	89.7	84.2	74.6	44.5	4	0	0
*2 g LD + 4 g q8h 4 h infusion*									*2 g LD + 4 g q6h 3 h infusion*								
60 mL/min	97.8	96.8	94.2	89.1	77.1	52.4	12.4	0.1	60 mL/min	99.4	99.1	97.7	96.2	91.1	74.5	27.4	0.5
90 mL/min	95.7	92.8	86.9	76.8	59.4	30.8	3.4	0	90 mL/min	98.7	97.5	95.8	91.3	78.8	55.1	12.7	0.3
120 mL/min	88.5	82.1	73.5	61.0	42.7	20.8	1.5	0	120 mL/min	96.7	93.1	88.5	78.9	64	37.7	6.1	0.1
150 mL/min	85	79.7	69.6	55.7	35.5	14.1	0.5	0	150 mL/min	93.4	89.7	84.2	75.6	57.9	30.4	4.3	0
*2 g LD + 8 g 24 h continuous infusion*									*2 g LD + 12 g 24 h continuous infusion*								
60 mL/min	100	100	100	100	100	88.1	22.1	0.1	60 mL/min	100	100	100	100	100	97.1	51.6	2.4
90 mL/min	100	100	100	100	99.6	73.5	8.4	0	90 mL/min	100	100	100	100	100	93.2	33.3	0.8
120 mL/min	100	100	100	100	98.2	52.1	3.9	0	120 mL/min	100	100	100	100	99.9	85.5	18.5	0.2
150 mL/min	100	100	100	99.9	95.7	41.6	1.8	0	150 mL/min	100	100	100	100	99.7	80.5	13.9	0
*2 g LD + 16 g 24 h continuous infusion*									*2 g LD + 22 g q6h 3 h infusion*								
60 mL/min	100	100	100	100	100	97.8	65.5	6.5	60 mL/min	98.7	98.7	98.7	98.7	98.7	96.5	69.6	13.5
90 mL/min	100	100	100	100	100	97.1	55.5	2.9	90 mL/min	99.5	99.5	99.5	99.5	99.5	96.8	68	8.5
120 mL/min	100	100	100	100	100	95.7	37.2	1.2	120 mL/min	100	100	100	100	100	97.2	59.6	4.2
150 mL/min	100	100	100	100	99.9	93.1	29.6	0.6	150 mL/min	100	100	100	100	100	96.9	51.4	2.6

^*a*^ CrCL, estimated creatinine clearance by Cockcroft-Gault; LD, loading dose; ƒ*C*_min_, unbound trough concentration; ƒ*C*_ss_, unbound average steady-state concentration in continuous infusion.

^*b*^ Highlighted in gray are the dosing strategies that attain a PTA ≥ 90% for a given target ƒ*C*_min_ or ƒ*C*_ss_ and CrCL.

**TABLE 4:** Dosing recommendations for empirical and targeted treatment stratified by estimated CrCL (Cockcroft-Gault) and target unbound trough (ƒ*C*_min_) or average steady-state concentration in continuous infusion (ƒ*C*_ss_)*^a^*^,^*^b^*

CrCL (mL/min)	Enterobacterales with high susceptibility Target ƒ*C*_min_ or ƒ*C*_ss_ ≥ 2 mg/L	Enterobacterales with intermediate susceptibility Target ƒ*C*_min_ or ƒ*C*_ss_ ≥ 4 mg/L	Empirical treatment/increased exposure for *Pseudomonas aeruginosa* Target ƒ*C*_min_ or ƒ*C*_ss_ ≥ 8–16 mg/L
**60–89**	2 g LD + 12 g daily continuous infusion or 2 g LD + 4 g q8h over a 4 h extended infusion	2 g LD + 12 g daily continuous infusion or 2 g LD + 4 g q6h over a 3 h extended infusion	2 g LD + 16 g daily continuous infusion or 2 g LD + 4 g q6h over a 3 h extended infusion^*d*^
**90–120**	2 g LD + 12 g daily continuous infusion^*c*^ or 2 g LD + 4 g q6h over a 3 h extended infusion		
**>120**	2 g LD + 12 g daily continuous infusion^*c*^		2 g LD + 16 g daily continuous infusion

^*a*^ The doses recommended provide the highest probability of attaining an optimized piperacillin exposure (defined as 100% ƒ*T*_>MIC_) with a lower total daily dose, considering an unbound toxicity threshold of ƒ*C*_min_ or ƒ*C*_ss_ ≥ 160 mg/L.

^*b*^ LD, loading dose.

^*c*^ Daily dose is lower for continuous infusion compared to extended infusion; continuous infusion provides a better attainment of 100% ƒ*T*_>MIC_ for these ranges of CrCL and should be considered for the treatment of serious infections.

^*d*^ Consider the higher dose for patients with highness sickness severity and if the epidemiology of the centre includes high-risk of less susceptible microorganisms.

When the more demanding PK/PD efficacy target was required (ƒ*T*_>4×MIC_), i.e., for the treatment of high-inoculum, deep-seated infections, simulations indicated that continuous infusion of piperacillin-tazobactam may be insufficient to achieve an optimal exposure for infections caused by less susceptible Enterobacterales (target ƒ*C*_min_ or ƒ*C*_ss_ ≥ 32 mg/L) or *P. aeruginosa* (target ƒ*C*_min_ or ƒ*C*_ss_ ≥ 64 mg/L), even when very high daily doses of ≥22 g were tested.

## DISCUSSION

To our knowledge, this is the largest multicenter study to describe the PK of piperacillin in hematological patients with febrile neutropenia. Our principal finding is that prolonged infusions (i.e., either extended or continuous infusions) improve early attainment of an effective exposure compared to short 30 min infusions. This is especially relevant in settings where achieving the PK/PD target is more challenging, either due to PK alterations such as increased Vd or augmented renal CL, or when more demanding exposures are necessary, i.e., for the empirical treatment of febrile neutropenia, where the risk of *P. aeruginosa* infections is high. These findings provide new evidence supporting the tailoring of β-lactam dosing in hematological patients, a group often underrepresented in PK studies but at high risk of early treatment failure.

Hematological patients with febrile neutropenia may exhibit significant alterations in the PK of hydrophilic drugs due to their specific pathophysiological characteristics (7). Our results are in agreement with this principle. In our cohort, the estimated Vd of piperacillin was approximately 30 L, nearly doubling the values reported in healthy volunteers (22) and consistent with previous findings, although calculated using different PK approaches, which also showed elevated Vd in patients with febrile neutropenia (26, 27). This expanded distribution may reflect third-spacing due to inflammation-related capillary leakage and fluid overload from the high-volume hydration administered during chemotherapy. This is a key finding, suggesting that, even in the absence of sepsis or septic shock, similar pathophysiological changes can alter drug distribution. Regarding CL, our population estimate for piperacillin was 12 L/h at a median CrCL of ~100 mL/min, aligning with previous studies in both healthy volunteers and febrile neutropenic patients (range: 5–15 L/h) (22, 26, 28) but different from others that report higher values (up to 18 L/h), highlighting the heterogeneity of this population (27, 29).

As expected, Cockcroft-Gault CrCL was the principal covariate that explained CL variability. Interestingly, we observed that 50% of our patients had a CrCL > 90 mL/min and 25% exceeded 120 mL/min with a mean 24 h diuresis of >2,000 mL, suggesting that the initial phase of febrile neutropenia may be characterized by a hyperdynamic state in the context of infection, fever, and fluid overload leading to augmented renal CL, similar to what has been reported in critically ill patients (30). This is particularly relevant for our patient population, in whom augmented renal CL should be suspected at the onset of febrile neutropenia, especially in those with preserved renal function.

In the context of these major alterations in PK, our dosing simulations show that the standard 4 g q6h regimen administered as a short 30 min infusion may be insufficient for treating *P. aeruginosa* infections, as this dose failed to provide optimal antibiotic exposure for isolates with an MIC ≥ 4 mg/L, the most likely scenario for susceptible *P. aeruginosa* (25). Furthermore, given the challenges in interpreting a single measurement of an MIC in the clinical setting, along with the rising antibiotic resistance among Gram-negative bacilli (31), targeting the clinical breakpoint (16 mg/L) may be justified for infections caused by *P. aeruginosa* (32) and for empirical treatment (33). In this context, prolonged infusions provided more stable drug concentrations over time and increased the likelihood of maintaining efficacious concentrations, as previously reported (26, 30, 34). Therefore, from a PK perspective, and in line with previous studies, the use of continuous infusions during the initial 24–48 h of therapy was shown to be especially beneficial, as this is the period when the most significant PK alterations occur, and the pathogen susceptibility is typically still unknown (26, 29). Continuous infusion could subsequently be maintained in patients with elevated CrCL, less susceptible pathogens, and/or high-inoculum deep-seated infections, such as nosocomial pneumonia (35). Once the etiological agent and its susceptibility to piperacillin-tazobactam are known, de-escalation strategies such as shorter infusions or a daily dose reduction (e.g., 12 g daily for Enterobacterales) may be considered for directed therapy based on the MIC and the ECOFF (32). In the scenario of high-inoculum infection, where a more demanding target of 100% ƒ*T*_>4×MIC_ should be considered, alternative therapeutic options or combination therapy with another anti-Gram-negative antibiotic should be considered if treating infections caused by less susceptible Enterobacterales or *P. aeruginosa* in this vulnerable population, as even continuous infusion of high doses of piperacillin (22 g daily) failed to provide optimal exposure for target ƒ*C*_min_ or ƒ*C*_ss_ ≥ 32–64 mg/L.

From a clinical perspective, however, the impact of prolonged infusions of β-lactam antibiotics on patients’ outcomes in febrile neutropenia remains uncertain (36), in contrast to patients with sepsis, where prolonged infusions have been associated with reduced all-cause mortality (37, 38). In febrile neutropenic patients, only two RCTs have compared extended versus short infusions of piperacillin-tazobactam with contradictory results regarding treatment success (11, 35) including the primary analysis of the BEATLE RCT, that did not find significant differences in treatment success and mortality between the two strategies (50.6% treatment success in extended infusion versus 63% in short infusion, *P* = 0.17) (11). These discrepancies arise in the context of the high clinical heterogeneity of febrile neutropenia during the empirical treatment phase, when a definitive diagnosis of infection is often not possible, only about 30% of episodes are microbiologically documented, and the etiology of fever is highly variable, including non-infectious fever. Nevertheless, prolonged infusions have shown a superior attainment of efficacious exposures and also a favorable safety profile, supporting their empirical use particularly in patients at risk of progressing to sepsis or of being infected with less susceptible microorganisms.

Our study has some limitations. First, only piperacillin total concentrations were measured, with unbound concentrations estimated assuming a theoretical protein binding of 30%. This assumption is considered reasonable, given that albumin concentrations were within the physiological range. In addition, concentrations of tazobactam were not measured. Previous studies have suggested that the % time above a threshold concentration is the PK/PD index most associated with tazobactam efficacy, classifying it as a time-dependent agent, despite the absence of intrinsic antibiotic activity and its lack of direct relationship with bacterial MIC (39). Furthermore, as with piperacillin, the PK of tazobactam is influenced by CrCL, and prolonged infusions are likely to enhance its exposure. Importantly, its efficacy has been shown to depend on the isolated microorganism and the expression of resistance mechanisms. Therefore, although current evidence suggests that prolonged infusions may improve its pharmacological profile, this warrants confirmation in future studies (26, 40). Second, patients with eGFR below 30 mL/min/1.73 m^2^ were excluded from the study, for which our recommendations do not apply to moderate to severe renal impairment. Third, most patients did not require admission to an intensive care unit, for which our findings cannot be extrapolated to critically ill patients with febrile neutropenia, nor to those with low-risk febrile neutropenia managed in outpatient settings. Conversely, the strengths of our research lie in its multicenter nature, the homogeneity of patients across treatment groups within a randomized clinical trial, and the comprehensive PK sampling performed on multiple dosing occasions over a treatment course, including intensive sampling during one of them. Moreover, dosing recommendations were based on both efficacy and toxicity thresholds, which is of particular importance for this patient group with multiple risk factors for nephrotoxicity and neurotoxicity (23, 24).

In conclusion, our findings suggest that prolonged infusions of piperacillin-tazobactam facilitate the achievement of optimal piperacillin exposures, particularly for patients with CrCL > 90 mL/min, and may be considered for the initial phase of the treatment of febrile neutropenia and for cases involving *P. aeruginosa* or other less susceptible Gram-negative bacilli.

## Data Availability

Individual data cannot be shared because of privacy restrictions. Raw anonymized data related to the PK analysis can be shared upon request with researchers who provide a methodologically reasonable proposal. The requests for data can be sent to the corresponding author. Interested researchers should obtain the approval of the Bellvitge University Hospital Ethics Committee.

## SUPPLEMENTAL MATERIAL

Supplemental Material aac.01253-25-s0001.docx Supplemental methods, Table S1, and Fig. S1.

ASM does not own the copyrights to Supplemental Material that may be linked to, or accessed through, an article. The authors have granted ASM a non-exclusive, world-wide license to publish the Supplemental Material files. Please contact the corresponding author directly for reuse.
