# Erratum for Laporte-Amargos et al., “Population pharmacokinetics and optimized dosing of piperacillin-tazobactam in hematological patients with febrile neutropenia”

**DOI:** 10.1128/aac.00740-26

**Published:** 2026-07-15

**Authors:** Julia Laporte-Amargos, Marta Ulldemolins, María Patricia Hernández-Mitre, Jason A. Roberts, Raul Rigo-Bonnin, Francisco Carmona-Torre, Maria Huguet, Pedro Puerta-Alcalde, Montserrat Arnan, Jose Luis del Pozo, Anna Torrent, Carolina García-Vidal, Anna Sureda, Alba Bergas, Enric Sastre-Escolà, Jordi Carratalà, Carlota Gudiol

## ERRATUM

Volume 70, no. 1, e01253-25, 2026, https://doi.org/10.1128/aac.01253-25. Table 3: “2 g LD +22 g q6h 3 h infusion” should read “2 g LD +22 g 24 h continuous infusion.”

