# Supplementary material for: Population pharmacokinetics and optimized dosing of piperacillin-tazobactam in hematological patients with febrile neutropenia
Source: Antimicrob Agents Chemother. 2025 Nov 25;70(1):e01253-25. doi: 10.1128/aac.01253-25 (PMC12777563; doi:10.1128/aac.01253-25)
Supplement: Supplemental Material — Supplemental methods, Table S1, and Fig. S1. [file aac.01253-25-s0001.docx]

**Supplementary material**

**Supplementary material on Methods**

Initially, one- and two-compartment models with first-order elimination were evaluated. The individual estimates for the PK parameters were assumed to follow a log-normal distribution. Between-subject variability (BSV) and between-occasion variability (BOV) were described using an exponential model according to the equation $\theta_{\mathrm{ik}}=\theta_{p}\times\exp\left( \eta_{i} \right)\times\exp\left( \eta_{\mathrm{ik}} \right)$, where $\theta_{\mathrm{ik}}$ is the estimated value of a parameter in the i^th^ patient and k^th^ occasion, $\theta$_p_ is the typical value of this parameter in the population, and η_i_ and η_ik_ are the individual deviations from the typical value representing the random BSV and BOV, which are assumed to follow a Gaussian distribution with a mean of zero and a variance of ω^2^ and γ^2^, respectively. The residual error was assumed to follow a standardised Gaussian distribution with a mean of zero and a standard deviation of one. Additive, proportional or combined (additive + proportional) error models were tested to describe residual variability. Model selection was based on the Akaike information criterion (AIC), the corrected Bayesian information criterion (BICc), the relative standard error (RSE) of the fixed and random effects and goodness-of-fit (GOF) plots.

From the base model, the influence of the following covariates on piperacillin PK parameters was explored: age, sex, height, weight at admission, eGFR (1) and CrCL estimated by the Cockcroft-Gault equation (2) at each sampling time, bilirubin plasma concentrations, albumin and total protein serum concentrations, and severity scores (Acute Physiology and Chronic Health Evaluation (APACHE) II (3), Sepsis-Related Organ Failure Assessment (SOFA) (4), and Multinational Association for Supportive Care in Cancer (MASCC) (5)). During the initial phase of the covariate analysis, the relationships between continuous covariates were assessed using correlation tests (Pearson or Spearman where appropriate) to identify potential collinearity. When collinearity was detected, the covariate with the higher correlation and greater biological plausibility was selected for inclusion in the model. For continuous covariates inclusion, covariates were log-transformed and normalised to their median values to linearise relationships with PK parameters and to normalise skewed distributions. Continuous and categorical covariates were individually added to the base model in the forward inclusion step until there was no drop in the -2xlog-likelihood (-2LL) over 3.84 (p < 0.05). In the backward elimination step, the covariates were removed from the model one by one, and the effect of each removal on the global fit was statistically tested. The covariates were kept in the final model if the backward elimination caused an increase in -2LL of at least 10.83 (p < 0.001) and the effect of the covariate on the PK parameter was biologically plausible.

**Model evaluation and internal validation**

Evaluation of the models was based on goodness-of-fit plots and prediction-corrected visual predictive checks (pc-VPC). GOF plots included observations versus individual predictions (IPRED) and population predictions (PRED), and plots of normalised prediction distribution error (NPDE) versus PRED and time after dose (6). A pc-VPC for each drug was performed using 500 simulations with the final models (7). Non-parametric bootstrapping (n=1000) performed in Monolix 2024R1 was used to assess the robustness of the three final models and to build confidence intervals for the parameter estimates.

**Supplementary material on Results**

**Table S1: Microbiology of microorganisms isolated in cultures during the study**

|  | N | MICⱡ (mg/L) |
| --- | --- | --- |
| **Bloodstream infection from abdominal source** |  |  |
| *Pseudomonas aeruginosa* | 2 | 4 |
| *Streptococcus mitis* | 2# |  |
| *Streptococcus salivarius* | 2# |  |
| *Escherichia coli* | 1# | 1 |
| *Klebsiella oxytoca* | 1# | 1 |
| *Klebsiella pneumoniae* | 1# | ≥ 128 |
| Methicillin-susceptible *Staphylococcus aureus* | 1# |  |
| *Bacteroides spp* | 1# |  |
|  |  |  |
| **Endogenous bloodstream infection** |  |  |
| *Pseudomonas aeruginosa* | 1 | 4 |
|  |  |  |
| **Urinary tract infection** |  |  |
| *Enterococcus faecalis* | 2 |  |
| *Escherichia coli* | 1* | ≤ 4 |
| *Klebsiella pneumoniae* | 1 | ≤ 8 |
| *Enterobacter cloacae* | 1 | 16 |
|  |  |  |
| **Stool culture** |  |  |
| *Campylobacter coli* | 1 |  |

ⱡ MIC: Minimum Inhibitory Concentration of the isolated microorganism.

N: number of isolates, corresponding to 13 patients.

# Three episodes were polymicrobial: *S. salivarius + S. mitis + S. aureus*, *E. coli + K. oxytoca*, *S. mitis* + *K. pneumoniae.*

* Positive blood and urinary cultures.

**Figure S1:** Goodness of fit plots for the final model. Upper figures: observed concentrations versus population (left) and individual (right) predictions for piperacillin plasma concentrations; the dotted red line represents the tendency line; the solid black line represents the identity line. Lower figures: Scatter plots of the normalised prediction distribution error (NPDE) over time in h and over population predictions for piperacillin concentrations (lower figures). The dotted red line represents the tendency line.


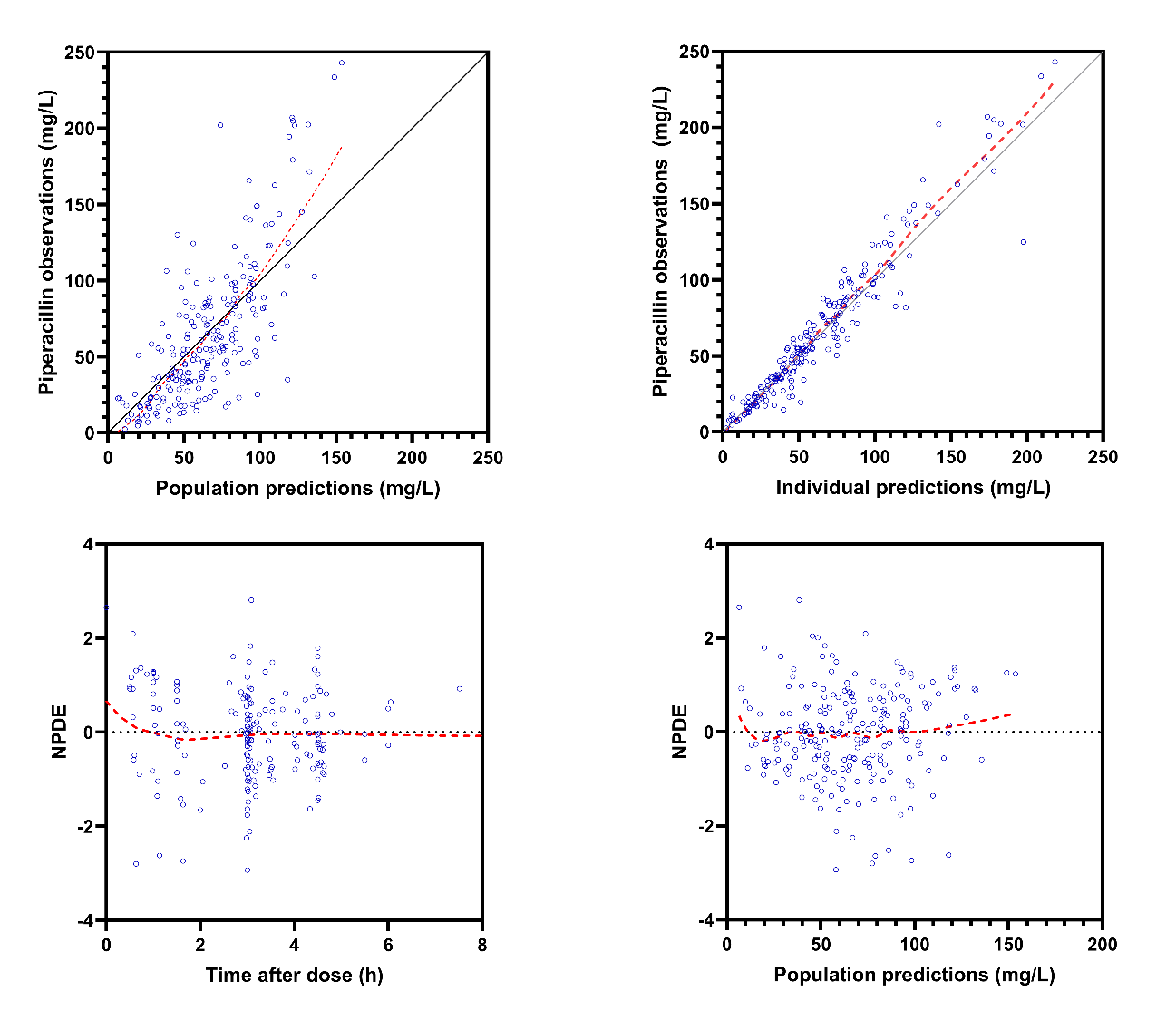


**References**

1. Levey AS, Stevens LA, Schmid CH, Zhang Y, Castro III AF, Feldman HI, Kusek JW, Eggers P, Van Lente F, Greene T, Coresh J. 2009. New equation to estimate glomerular filtration rate? Ann Intern Med 150:604–612.

2. Cockcroft DW, Gault MH. 1976. Prediction of creatinine clearance from serum creatinine. Nephron 16:31–41.

3. Knaus WA, Draper EA, Wagner DP, Zimmerman JE. 1985. APACHE II: a severity of disease classification system. Crit Care Med 13:818–829.

4. Vincent J-L, Moreno R, Takala J, Willatts S, De Mendonça A, Bruining H, Reinhart CK, Suter PM, Thijs LG. 1996. The SOFA (Sepsis-related Organ Failure Assessment) score to describe organ dysfunction/failure. Intensive Care Med 22:707–710.

5. Klastersky J, Paesmans M, Rubenstein EB, Boyer M, Elting L, Feld R, Gallagher J, Herrstedt J, Rapoport B, Rolston K, Talcott J. 2000. The Multinational Association for Supportive Care in Cancer Risk Index: A Multinational Scoring System for Identifying Low-Risk Febrile Neutropenic Cancer Patients. J Clin Oncol 18:3038–3051.

6. Mentré F, Escolano S. 2006. Prediction discrepancies for the evaluation of nonlinear mixed-effects models. J Pharmacokinet Pharmacodyn 33:345–367.

7. Bergstrand M, Hooker AC, Wallin JE, Karlsson MO. 2011. Prediction-corrected visual predictive checks for diagnosing nonlinear mixed-effects models. AAPS J 13:143–151.
